# HER4 Promotes Osteosarcoma Progression and Predicts Poor Prognosis through the PTEN-PI3K/AKT Pathway: Retraction

**DOI:** 10.7150/jca.110321

**Published:** 2025-01-16

**Authors:** Kun Ma, Chuan Zhang

**Affiliations:** Luoyang Orthopaedic Hospital of Henan Province & Orthopaedic Hospital of Henan Province, Luoyang, Henan 471002, China.

We, the authors, would like to retract our paper entitled “HER4 Promotes Osteosarcoma Progression and Predicts Poor Prognosis through the PTEN-PI3K/AKT Pathway” due to concerns on image duplications. We would like to express apology for our negligence and mistakes in this paper.

